# Molecular Dynamics Simulation of Ligand Dissociation from Liver Fatty Acid Binding Protein

**DOI:** 10.1371/journal.pone.0006081

**Published:** 2009-06-30

**Authors:** Dong Long, Yuguang Mu, Daiwen Yang

**Affiliations:** 1 Department of Biological Sciences, National University of Singapore, Singapore, Singapore; 2 School of Biological Sciences, Nanyang Technological University, Singapore, Singapore; University of Cincinnati, United States of America

## Abstract

The mechanisms of how ligands enter and leave the binding cavity of fatty acid binding proteins (FABPs) have been a puzzling question over decades. Liver fatty acid binding protein (LFABP) is a unique family member which accommodates two molecules of fatty acids in its cavity and exhibits the capability of interacting with a variety of ligands with different chemical structures and properties. Investigating the ligand dissociation processes of LFABP is thus a quite interesting topic, which however is rather difficult for both experimental approaches and ordinary simulation strategies. In the current study, random expulsion molecular dynamics simulation, which accelerates ligand motions for rapid dissociation, was used to explore the potential egress routes of ligands from LFABP. The results showed that the previously hypothesized “portal region” could be readily used for the dissociation of ligands at both the low affinity site and the high affinity site. Besides, one alternative portal was shown to be highly favorable for ligand egress from the high affinity site and be related to the unique structural feature of LFABP. This result lends strong support to the hypothesis from the previous NMR exchange studies, which in turn indicates an important role for this alternative portal. Another less favored potential portal located near the N-terminal end was also identified. Identification of the dissociation pathways will allow further mechanistic understanding of fatty acid uptake and release by computational and/or experimental techniques.

## Introduction

Liver fatty acid binding protein (LFABP) belongs to the fatty acid binding protein (FABP) family which accommodate poorly soluble ligands in a β-barrel cavity and maintain their solubility during intracellular transportation [1]. Although the first three-dimensional FABP structure was solved by crystallography two decades ago [2], the mechanism of how fatty acids access the binding sites, which locate inside the protein cavity, still remains largely unknown up to date, since the crystal structure shows no obvious openings on the protein surface which could allow the entry and exit of ligands. Furthermore, uniquely in the FABP family, LFABP is known to accommodate two fatty acids as well as other bulky and rigid ligands such as fatty acid-CoA thioesters, lysophosphatidic acid, bile salts, heme, 1,8-ANS, lipophilic drugs etc. [1], [3], [4]. Thus, it is particularly interesting to know how ligands access the binding cavity.

The three-dimensional structure of LFABP was first solved using X-ray crystallography [5], and showed a similar overall conformation with other FABPs. Two molecules of bound oleates were found inside the protein cavity, and the second binding site seemingly occupied a channel leading to the bulk solvent [5]. The fact that the carboxylate group of the second oleate is in a solvent-exposed position near the hypothetical “portal region” supports the hypothesis that the ligands exchange with the exterior environment through the area delimited by α-helix II, βC/βD loop, and βE-βF loop. This “portal region” has been investigated by numerous studies [6]–[10], and seems to be common for the family members. On the other hand, different “alternative portals” were also hypothesized for several family members [2], [11]–[13]. Considering the unique binding stoichiometry of LFABP, tentative “alternative portals” could possibly exist for special functions.

Due to the dynamical nature of ligand dissociation processes, mere structural studies are insufficient to reveal the dissociation pathways. And the transient and intricate nature of this dynamical process renders it very difficult for experimental investigation. Therefore molecular dynamics (MD) simulation has been extensively used to explore the dynamics of FABPs as well as their interaction with fatty acids [11], [12], [14]–[19]. However, dissociation of fatty acids from FABPs takes place on a timescale of seconds [20], which is beyond the accessible simulation time of ordinary MD simulations. Thus, complete entry or exit processes were rarely observed in ordinary MD simulations. Tsfadia and coworkers [17] recently reported a successful case of MD simulation in which a palmitate successfully penetrated into the cavity of toad liver basic fatty acid binding protein (Lb-FABP) from the bulk solution, which, nonetheless, was quite infrequent in the extensive simulations of many independent runs. Dissociation of the fatty acid from intestinal FABP (IFABP), which was facilitated by a predefined force with constant direction, was also reported [12]. However, steered simulation, though being very effective in dissociating the ligand, could not be used to identify tentative new pathways.

In the current study, we aim at an extensive unbiased search of tentative dissociation pathways of ligands from LFABP. Random expulsion molecular dynamics (REMD) simulation [21]–[23], which applies a randomly oriented force to accelerate the dissociation process, is particularly suited for the current purpose. Using this method, we have thoroughly examined the dissociation pathways of different ligands from LFABP and revealed alternative portals, in addition to the common “portal region”. One alternative portal from our simulation result agrees well with the previously hypothesized alternative portal for LFABP. Our comparative studies with IFABP also indicate that this alternative portal is related to the unique structural feature of LFABP.

## Methods

### Molecular dynamics simulation

The initial crystal structures of LFABP-oleate complex and IFABP-palmitate complex were obtained from the Protein Data Bank (PDB ID: 1LFO and 2IFB respectively). The initial structure of LFABP complexes with two ANS molecules was obtained from the molecular docking results [3]; only one of three representative poses of ANS at the high affinity binding site was used in the current study. The AMBER-03 all-atom force field [24] was used to model the protein molecules. The partial atomic charges of oleate (OLA), palmitate (PLM), and 1-anilino-8-naphthalene sulfonate (1,8-ANS) were calculated following the AMBER convention [25]. Other force field parameters for these ligands were assigned in analogy to the existing force field. Neutralization of the oleate anion was done by adding +0.5*e* to the assigned charge of each oxygen atom. Explicit water molecules, described using the TIP3P model [26], were filled in the periodic cubic box for the all atom simulation, and the system was neutralized by adding Na^+^ or Cl^−^ ions. The electrostatic interactions were treated using the fast particle-mesh Ewald summation method [27]. And the temperature during simulation was kept constant at 300 K by Berendsen's coupling [28]. GROMACS software package [29] was used to perform the simulation with a time step of 2 fs. Prior to REMD simulations, the initial structures were relaxed by energy minimization using steepest descent algorithm, followed by 100 ps equilibration with a harmonic potential restraint applied to all the heavy atoms of the protein-ligand complexes. Another 100 ps unrestrained simulation was carried out for LFABP-oleate complex and IFABP-palmitate complex as further equilibration; 1 ns unrestrained simulation was carried out for the LFABP-ANS complex. The systems were then subjected to REMD simulations.

### Parameters for random expulsion simulation

In this study, a computational protocol, slightly modified from Ludemann and coworkers' method [22], was used for REMD simulations. An additional acceleration ( ***a*** ) with constant amplitude (2∼4 nm/ps^2^) and a randomly chosen direction was applied to every atom of the ligand; 2 nm/ps^2^ acceleration of OLA, PLM and 1,8-ANS corresponds to the force constants (by Ludemann *et al*. 2000) of 563, 511 and 597 kJ/mol/nm, respectively. During the short time interval (Δt = 0.25 ps), the direction was kept constant. The displacement ( ***r*** ) of the center of mass of a ligand was calculated for this time interval. If the dot product of the displacement and the normalized acceleration, ( *l* = ***r*** **·** ***a/|a|*** ), is above a predefined threshold ( *l*_min_ ), the direction of the acceleration for the next Δt interval will be updated to the direction of the displacement; otherwise, ***a*** is updated with a randomly chosen direction. Each REMD simulation was run for a maximum of 200 ps; it would automatically stop once the ligand dissociated away from the protein by 0.8 nm or the simulation time reached the maximum time of 200 ps. For the REMD simulation of holo-LFABP bound with two molecules of the ligands (OLA or 1,8-ANS), the random acceleration, unless otherwise stated, was firstly applied to the ligand at the low affinity site alone, and shifted to the another ligand after the first one had dissociated away from the protein.

### Identification of residues constituting the portals

Residues, which are in close contact with the ligand molecules during the dissociation courses, are considered to be key residues constituting individual portals. The backbone nitrogen atoms of three residues spanning the individual portal regions were used to define a reference plane. When each non-terminal carbon atom of the fatty acids (C5–C14 for OLA; C4–C13 for PLM) was passing through the portals (its distance to the reference plane was within 0.1 nm), the minimum distance between this carbon atom and each atom of a residue was calculated. If any atom of a given residue is within the distance cut-off of 0.5 nm, this residue was recorded as a potential portal residue. Totally 45 and 36 trajectories (last 10 ps) were analyzed for LFABP and IFABP, respectively.

## Results and Discussion

### Dissociation of OLA128 from holo-LFABP

**Figure 1:**
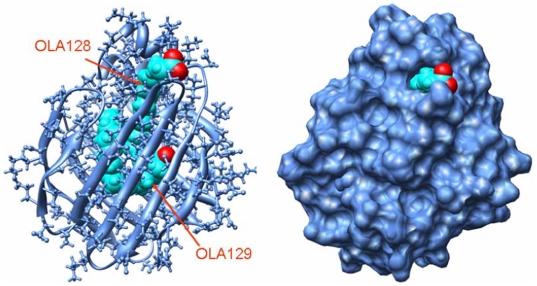
Three-dimensional structure of LFABP-oleate complex. The protein structure is shown in ribbon (left) and surface (right) representations; oleic acid is displayed in a sphere representation. Hydrogen atoms were added from the crystal structure (pdb ID: 1LFO) using GROMACS software; energy was minimized using steepest descent algorithm to remove bad contacts. The structures are displayed using UCSF Chimera.

**Figure 2:**
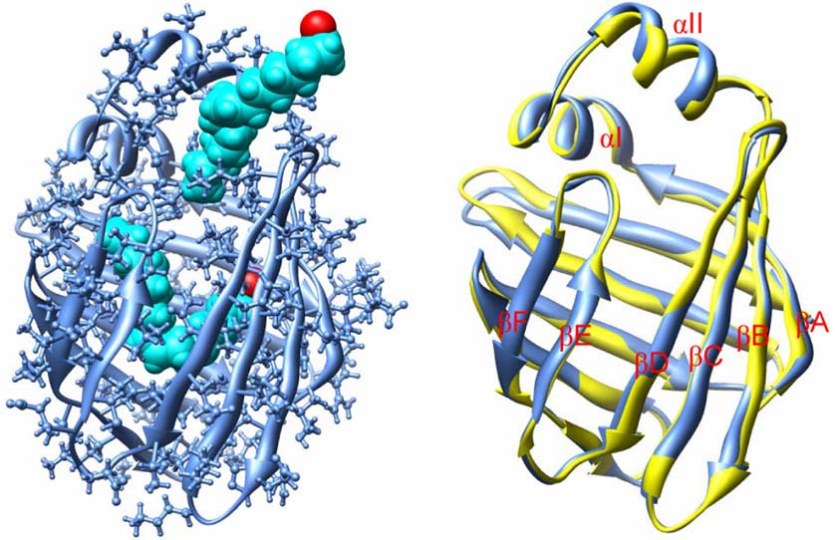
Dissociation of OLA128. A snapshot taken during the egress of OLA128 from the cavity of LFABP (left); alignment of the backbone conformation (shown in the right panel; painted blue) with the starting conformation of REMD (painted yellow). Oleate molecules are shown in cyan; oxygen atoms of OLAs are shown in red.

As shown in the X-ray structure of the LFABP-oleate complex, the carboxylate group of OLA128 (Figure 1) is protruding outside the β-cavity from the area delimited by α-helix II, βC/βD loop, which roughly agrees with the hypothetical “portal region” of other FABPs [1]. Thus, egress of OLA128 through this region was supposed to be the most likely route. Ten independent REMD runs were carried out to dissociate OLA128 using a random acceleration of 2 nm/ps^2^, and all of these independent results showed the successful egress of OLA128 in a short time. As expected, the egress in all runs took place through the “portal region”. A representative conformation of the protein-OLA complex, in which OLA128 was about to leaving the protein, is shown in Figure 2. In the course of the OLA128 dissociation, small changes in the protein backbone conformation appeared to happen; the rotation of the sidechains of a few residues further enlarged the opening size so as to allow the egress of OLA128. The result indicates that large conformational re-organization of the protein should not be the necessity for the dissociation of oleate molecules from this path.

### Dissociation of OLA129 from holo-LFABP

After OLA128 dissociated from the protein, the second ligand (OLA129) was still bound in an inner position of the protein cavity, which was distant from the “portal region”. Thus, there were no intuitive suggestions of how it would dissociate from the protein. In the initial five trials of dissociating the second ligand (OLA129) using the same REMD parameters with those for OLA128, the exit process failed to occur in a 200 ps time limit, which was mainly due to the tight interaction between OLA129 and Arg122. In order to effectively dissociate OLA129, we increased the amplitude of the random acceleration to 3 and 4 nm/ps^2^, respectively For each value of the random acceleration, fifteen independent runs were carried out to unbind OLA129. Egress of OLA129 was observed in all the thirty runs, and unexpectedly, three different egress routes were found for OLA129 (Table 1 & Table 2).

**Table 1:** REMD simulation of ligand exiting from the cavity of IFABP and LFABP.

	Ligand	| *a* | (nm/ps^2^)	*l*_min_ (nm)	Portal I†,**	Portal II†	Portal III†	other	Failed to dissociate‡
LFABP	OLA128	2	0.015	10	0	0	0	0
	OLA129	2	0.015	0	0	0	0	5
	OLA129	3§	0.030	3	11	1	0	0
	OLA129	4§	0.050	3	12	0	0	0
	OLA129n	2§	0.015	5	7	3	0	0
	OLA129#	3	0.030	0	14	0	1*	0
	ANS128	2	0.015	9	1	0	0	0
	ANS129	2	0.015	6	5	2	0	2
IFABP	PLM	2§§	0.015	16	0	0	0	4
		3§§	0.030	20	0	0	0	0

The time interval (Δt) of REMD was kept constant at 0.25 ps for all trajectories.

# Random force was applied on OLA129 only, while OLA128 was present in the low affinity binding site.

† The number of trajectories in which the ligand successfully egressed using the individual portals.

‡ The number of trajectories in which the ligand failed to exit from the cavity in 200 ps.

** For IFABP, portal I refers to the helical portal.

* In this single trajectory, OLA129 partially protruded outside from portal II initially, then tried to slide over βE/βF loop, and eventually dissociated from the gap between the βE/βF loop and α-helix cap.

§ Trajectories were used for the analysis of residues constituting individual portals of LFABP (Table 2).

§§ Trajectories of successful dissociations were used for the analysis of residues constituting the portal of IFABP (Table 2).

**Table 2:** Residues constituting the portal regions.

		Reference residues§	Residues#
LFABP	Portal I	28, 31, 56	28,31,32,35,54,55,56,57
	Portal II	22, 77, 96	18,22,24,73,74,75,77,79,95,96,98,115
	Portal III	45, 88, 106	1,3,43,65,85,91,104
IFABP	Helical portal	30, 55, 73	14,18,23,27,28,30,31,34,55,72,73,74

§ Coordinates of the backbone nitrogen atoms of these residues determined the reference plane.

# Residues constituting each portal were determined as described in the Methods section. Totally 11, 30, 4 and 36 trajectories were used for the analysis of portal I, portal II, portal III of LFABP and the helical portal of IFABP, respectively. For portals I and III of LFABP, only the residues which were recorded as potential portal residues in at least two different trajectories were finally reported; for portal II of LFABP and the helical portal of IFABP, only the residues which were recorded in at least four different trajectories were finally reported.

**Figure 3:**
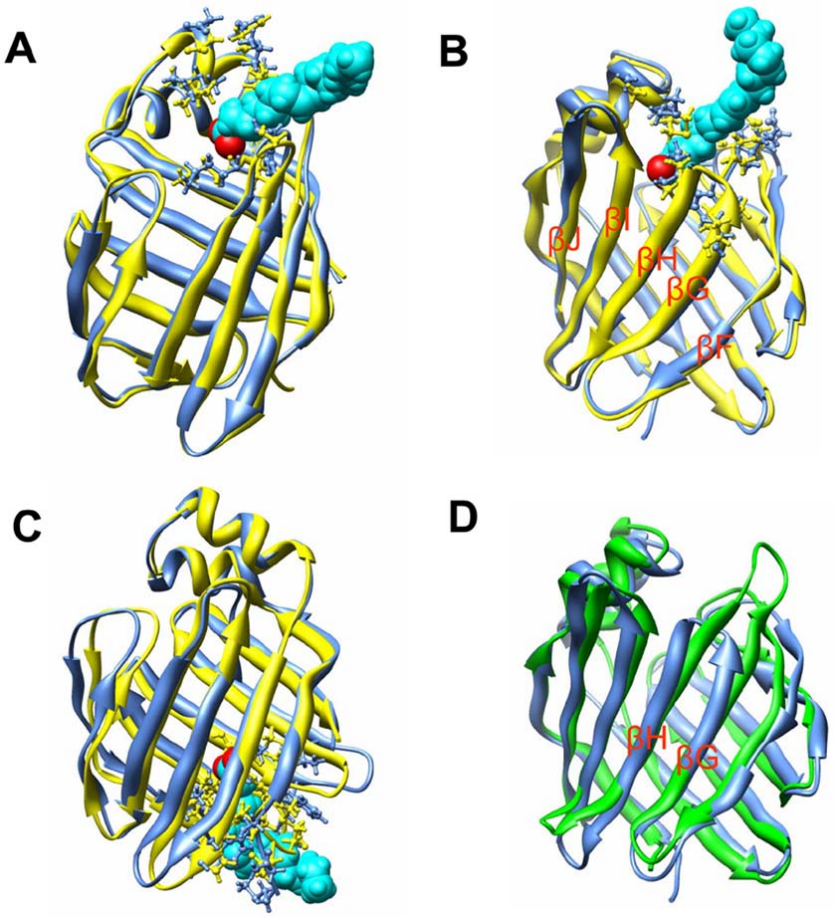
Dissociation of OLA129. (A–C) Snapshots showing three different dissociation processes of OLA129 by portal I, II and III, respectively; these three representative trajectories are also used for the calculation of RMSF (shown in Figure 5). The starting conformation (yellow) is aligned with the protein conformations in the moment of ligand expulsions (blue). The sidechains of residues which are surrounding OLA129 are shown in the ball-and-stick representation. (D) Structural alignment of rat-LFABP (blue) and rat-IFABP (green). βH and βG strands of IFABP are significantly longer than those of LFABP.

Although the “portal region” delimited by α-helix II and βC/βD loop (denoted as portal I in this article) could still be used for OLA129 to exit the cavity (Figure 3*A*), it was not the sole choice anymore and seemed not even to be the primary choice. As shown in Table 1, in three out of the fifteen runs at the acceleration of 3 nm/ps^2^, OLA129 egressed from portal I. By contrast, in eleven runs the OLA129 left the protein from an alternative region delimited by the βG/βH loop, βE/βF loop, C-terminal end of α-helix I, and N-terminal end of αI/αII loop, which is denoted as portal II in this article (Figure 3*B*). Although portal I was extensively studied, portal II was very rarely mentioned in literature. However, there are a few evidences indicating that portal II could be a unique portal for LFABP. The structural comparisons between LFABP and other intracellular lipid binding proteins (iLBPs) revealed that the βG and βH strands of LFABP are two residues shorter than the average of other iLBPs [5], which provides the structural basis of forming an opening at the region of portal II (Figure 3*D*). Furthermore, in the exchange studies of LFABP with ^13^C-labeled fatty acids [13], OLA129 was proposed to be involved in direct exchange with either OLA128 or the oleate molecules in the bulk solvent without displacing OLA128. Both of these two possibilities seemed quite unlikely to happen intuitively, but provided portal II could be used for OLA129 to exit the cavity, the second explanation would become understandable [13]. However, on the basis of Wang and coworkers' NMR studies, there is no evidence showing portal II could be used as a dissociation pathway. In our current REMD simulation, portal II was frequently used by OLA129 to exit the LFABP cavity, which should be the first direct evidence supporting this hypothesis.

Besides portal I and portal II, there was one case in the 15 runs (with acceleration of 3 nm/ps^2^), in which OLA129 dissociated from the protein via the bottom of the cavity (Figure 3*C*), denoted as portal III in this article. The possibility of ligand penetration from the bottom of adipocyte lipid binding protein (ALBP) was previous discussed [11]. In this study, we showed that ligand exit from the bottom of the cavity was also possible for LFABP. However, this region was the least frequently used one for OLA129 to dissociate. In addition, at a larger amplitude of acceleration (4 nm/ps^2^), the egress of OLA129 from portal III was not observed. Thus, portal III is regarded as the least likely portal here.

### Residues constituting individual portals

**Figure 4:**
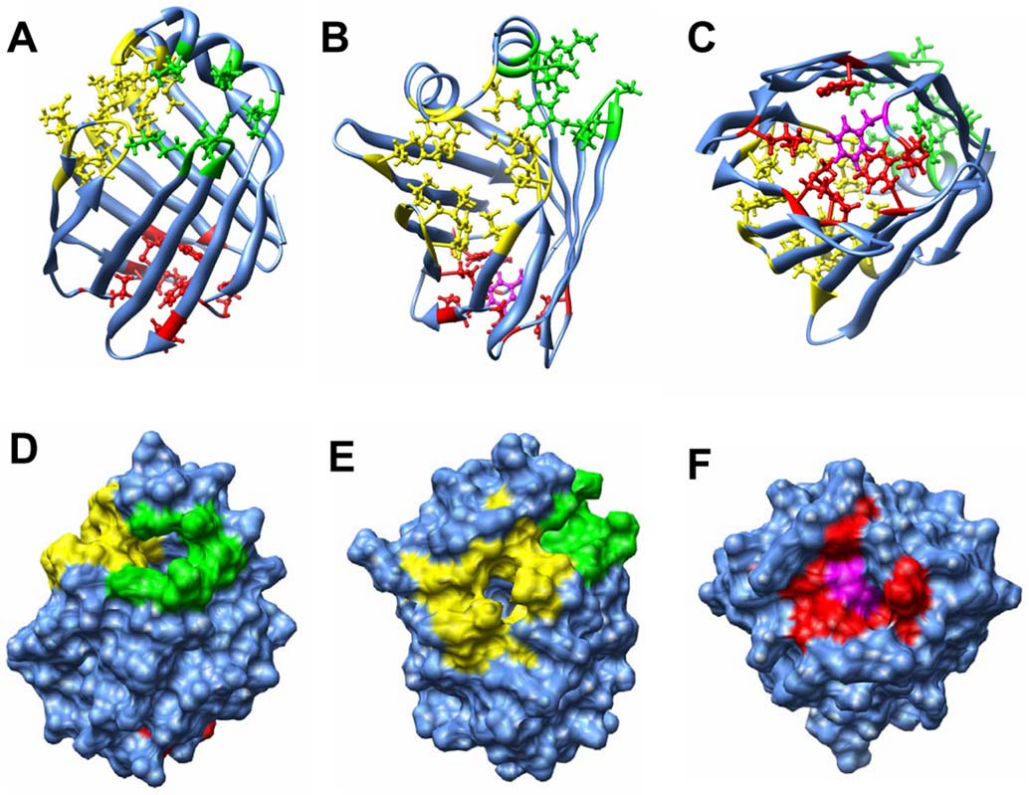
Front views of three portals. Three portal areas in the starting structure of REMD (time at zero) are shown in ball-and-stick (A–C) and surface (D–F) representations. Residues constituting portal I, II, and III are colored green, yellow, and red, respectively. Phe3 is colored pink (in C and F). Oleate molecules are not shown in the structure.

Residues which were commonly encountered by the ligand during expulsion should be important for the constitution of individual portals. Constituting residues, identified as described in the methods section, are listed in Table 2. These residues are highlighted on the starting protein structure of our REMD simulations (Figure 4). Small openings at the portal I and portal II regions could form even before the application of the external force, which indicated the potentiality of ligand dissociation from these regions. Portal III was closed in the starting structure, however, certain parts of portal III (e.g. Phe3 at the N-terminal loop; colored pink in Figure 4) were supposed to be plastic, showing the potentiality of creating an opening at this region. Nevertheless, untying the side-chain packing of hydrophobic residues at this region would not be a particularly easy process, thus this portal was the least recorded one in multiple repetitions of REMD simulations.

In order to test whether the same egress routes (portal I–III) could be observed at lower amplitudes of acceleration, another set of REMD simulations (15 runs) were set up (with ***a*** = 2 nm/ps^2^). Since the formation of the salt bridge was identified as the time-consuming step, which kept OLA129 inside the cavity in 200 ps time in our initial trials using the same value of acceleration, the neutralized OLA molecule (OLA129n) was used for these simulations. As shown in Table 1, egress from all three portals was observed, which demonstrates the plausibility of the three portals. These 15 trajectories were also included in the analysis of portal residues.

### Root mean squared fluctuations (RMSF) during the dissociations

**Figure 5:**
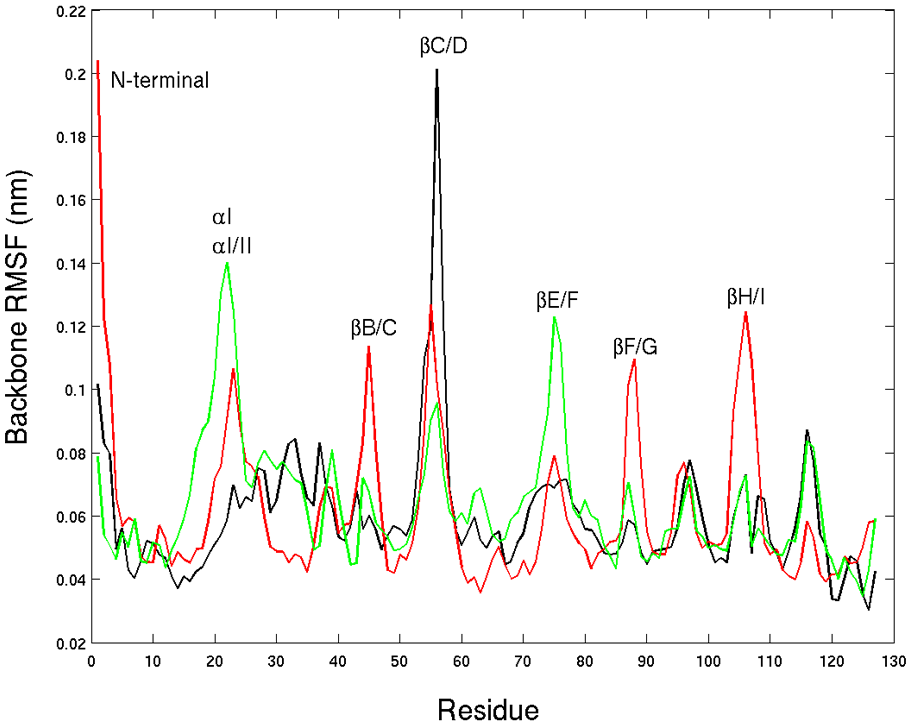
Backbone residue-wise root mean square fluctuations (RMSF) of LFABP. The backbone RMSF of LFABP for the dissociation processes from portal I (black), portal II (green), and portal III (red) are plotted against the residue number; calculations are based on the last 10 ps, 13.5 ps (which is the whole trajectory) and last 15 ps of trajectories for portals I, II and III, respectively.

Comparison of protein backbone conformational fluctuations during the dissociation from the three portals is shown in Figure 5. For the trajectories in which OLA129 exit from portal I and portal III (the black and red curves in Figure 5 respectively), the ligand initially attempted to dissociate from other portals during the random expulsions, but eventually exited from portal I or III respectively. In order to minimize the RMSF caused by these initial attempts, only the last 10 ps and 15 ps of the respective trajectories were used for analysis. As expected, most significant conformational fluctuation took place at the regions close to the individual portal. For the red curve (corresponding to portal III), the increase of RMSF at the α-helix region and βC/βD loop is believed to be caused by the residual effects of initial attempts to exit from these regions. Regions with increased RMSF, directly contributed by ligand exiting from portal III, are N-terminal loop, βB/βC loop, βE/βF loop, and βH/βI loop, which surround the bottom of the cavity. Clearly, dissociation from this portal requires protein conformational changes in much larger areas than portal I and II, which possibly explains why it is the least preferred route in our multiple independent runs.

### Which portal does OLA129 dissociate from when OLA128 still binds the protein?

**Figure 6:**
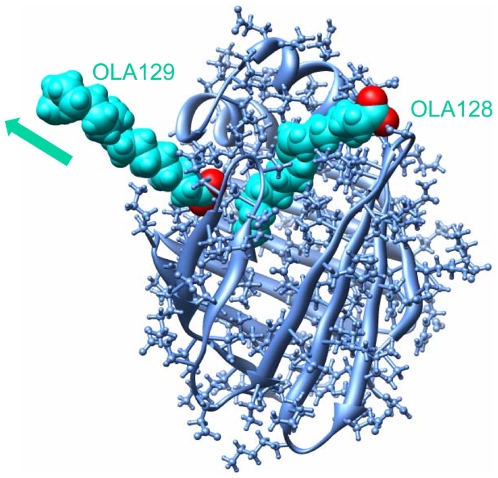
Dissociation of OLA129 from portal II without displacing OLA128.

In the X-ray structure of LFABP-OLA complex [5], OLA128 occupied the channel connecting the portal I region and OLA129. Thus, if portal I is the only choice for exiting the cavity, it appears to be a necessity that OLA128 must come out first. However, an alternative portal (portal II) might allow the direct exchange between OLA129 and bulk OLA [5], [13]. Based on the fatty acid exchange studies [13], OLA129 was assumed to be involved in exchange with OLA128 within the cavity or direct exchange with bulk OLA without displacing OLA128. This latter possibility was discussed in details [13], but there was no direct evidence showing the feasibility of such a process. In order to evaluate the feasibility of the structural dynamics underlying this hypothesis, random acceleration was directly applied to only OLA129 in the doubly ligated protein complex. In the fourteen independent runs, successful egress of OLA129 from portal II was observed (Figure 6). In one exceptional case among the 15 runs (Table 1), OLA129 partially protruded out from portal II initially, then tried to slide over βE/F loop, eventually dissociated from the gap between βE/F loop and α-helix cap. This deviation from the typical portal II region was caused by the applied external acceleration which adjusted away its direction during egress, thus it is regarded as an artifact, and not considered any more here. Our current results show that conformational adjustment of LFABP and OLA128, allowing OLA129 to egress without displacing OLA128, is feasible. Furthermore, although three different portals could be used by OLA129 in the absence of OLA128, portal II becomes the dominating route in the presence of OLA128.

### Dissociation of 1,8-ANS from LFABP

Long chain fatty acids, which are known as the natural ligands for LFABP, are linear molecules with significant flexibility; adjustment of the fatty acid conformation during the egress could be easily done to fit the openings created. A number of compounds with rigid ring structures were recently identified as the ligands of LFABP [3]. Thus whether rigid ligands can egress from the same routes for fatty acid is of significant interest. 1,8-ANS is widely used as the fluorescent probe for studying the interaction of ligands with FABPs [3], [30], [31]. Since two ANS molecules bind in analogy to the positions of the corresponding oleate molecules, they are named ANS128 and ANS129 respectively in this study. As ANS128 was close to portal I, egress from portal I would be the most favorable route. In nine out of ten independent runs (Table 1), ANS128 dissociated from portal I as expected; while in one case of the simulations, ANS128 was dragged by the random force to a much inner position and eventually dissociated from portal II. Under normal conditions without any external forces, such a situation is unlikely to happen, thus portal I is believed to be the only choice for ANS128.

**Figure 7:**
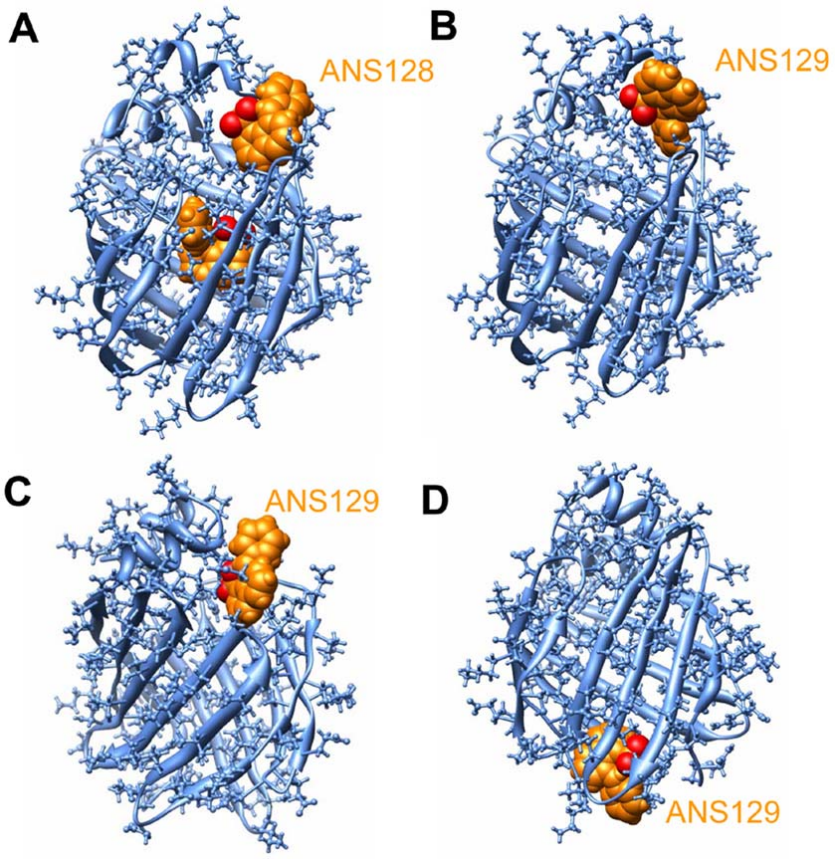
Snapshots of 1,8-ANS exiting the cavity. (A) ANS128 exiting from portal I. (B–D) ANS129 exiting from portal I, portal II and portal III, respectively. 1,8-ANS is displayed in a sphere representation (orange).

ANS129 did not form stable electrostatic interaction with LFABP in the random expulsion, but it appeared to be generally more difficult to penetrate out of the protein surface than OLA129n, which is attributed to the rigidity of the ANS molecule. Random updates of the acceleration generally occurred more frequently; in two out of fifteen runs, ANS129 failed to egress within 200 ps. However, when it approached the portals in favorable poses, it could readily egress from three individual portals (Table 1, Figure 7). This result demonstrates that the three portals, previously identified on OLA, can also be utilized by rigid ligands.

### Comparative study between intestinal FABP and liver FABP

Although IFABP and LFABP share a quite similar folding pattern, portal II seems not to exist in IFABP (Figure 3*D*). Release of fatty acid through the helical portal region and β-strand portal (which roughly correspond to portal I and portal III, respectively) was discussed previously [12]. In the steered simulation [12], release of palmitate through both regions occurred, and the helical portal was found to be more favorable in terms of energy cost and conformational changes.

**Figure 8:**
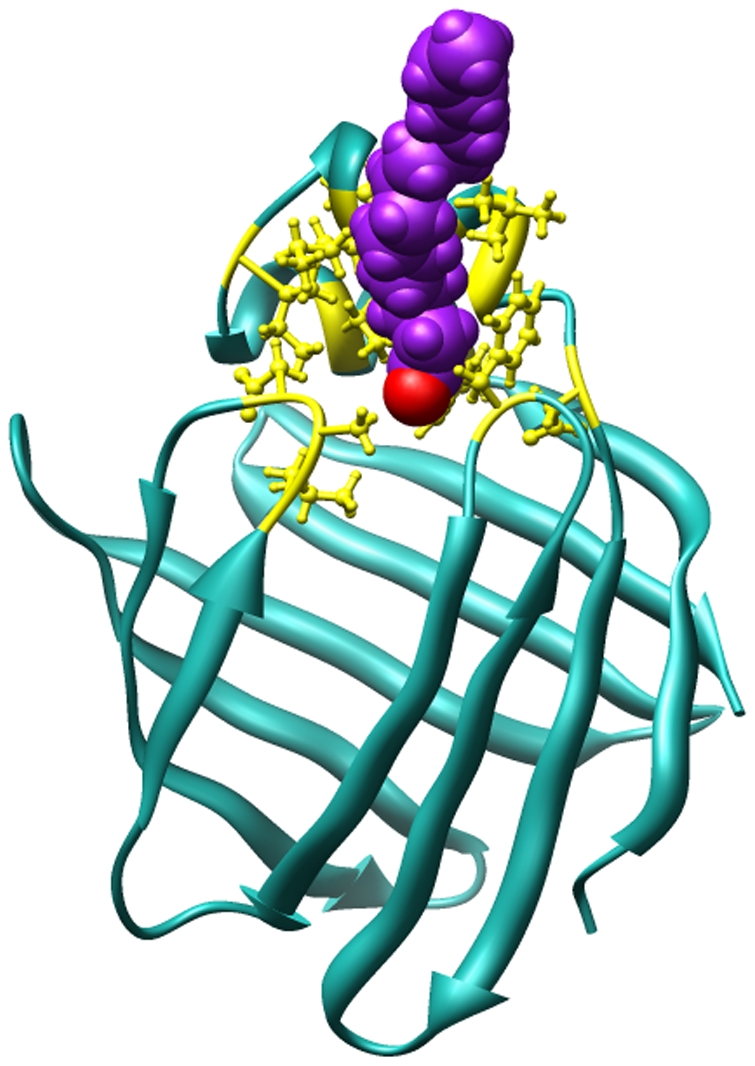
Dissociation of palmitate (purple) from the cavity of IFABP (dark green). Residues constituting the helical portal (Table 2) are shown in yellow. In all the runs of successful dissociations, the palmitate molecule came out from essentially the same region.

Since three different portals were found for LFABP, it is quite interesting to know whether different portals could also be found for IFABP in the REMD simulation. In total, we conducted 40 independent REMD runs (with ***a*** values of 2 or 3 nm/ps^2^) to dissociate the bound palmitate from IFABP. Quite different from the situations of LFABP, in all the successful dissociations, the helical portal of IFABP was found to be the only possible route (Table 1, Figure 8). This result showed the uniqueness of the alternative portal(s) for LFABP. In addition, the helical portal of IFABP, although having a roughly similar location with portal I of LFABP, showed a slight difference from the latter. The βE/βF loop, which was rarely observed to contact dissociating ligands from LFABP (Figure 4A, Table 2), was shown to be a major constituting part for the helical portal of IFABP (Figure 8).

### General conclusions

The ligand binding sites of FABPs, shown by X-ray crystallographic studies, have been known over decades. However, the mechanism of how the ligands access their binding sites remains unresolved up to date, mainly due to the limitations in the methods/techniques which can study this process. In this study, we aim at an extensive search of the possible ligand-escaping routes for LFABP that exhibits substantial complexity and variety in its interactions with ligands. Although an external force (in the form of a random acceleration) was applied in our simulation, the random nature of this force ensured the objectivity of this study, at least to an acceptable level. Multiple repetitions of the simulation and comparative studies over different ligands and different proteins showed the reliability of our current results.

In conclusion, two alternative portals, besides the primary portal, were identified for LFABP in this work. Portal II was shown to be a highly preferred region for OLA129 to dissociate, which is probably the major portal for the inner bound ligand under physiological conditions. Comparative studies with IFABP showed that formation of portal II should be uniquely related to the structural feature of LFABP. In addition, we also showed the possible existence of another portal (portal III). However, this portal does not seem to be favorable for ligand dissociation. Further investigation of this region using other experimental/theoretical approaches would be an interesting future direction.
